# Evaluation of Sheep Anticipatory Response to a Food Reward by Means of Functional Near-Infrared Spectroscopy

**DOI:** 10.3390/ani9010011

**Published:** 2018-12-29

**Authors:** Matteo Chincarini, Lina Qiu, Lorenzo Spinelli, Alessandro Torricelli, Michela Minero, Emanuela Dalla Costa, Massimo Mariscoli, Nicola Ferri, Melania Giammarco, Giorgio Vignola

**Affiliations:** 1 Università degli Studi di Teramo, Facoltà di Medicina Veterinaria, Località Piano d’Accio S.P. 18, 64100 Teramo, Italy; mmariscoli@unite.it (M.M.); mgiammarco@unite.it (M.G.); gvignola@unite.it (G.V.); 2 Politecnico di Milano, Dipartimento di Fisica, piazza Leonardo da Vinci 32, 20133 Milano, Italy; lina.qiu@polimi.it (L.Q.), alessandro.torricelli@polimi.it (A.T.); 3 Consiglio Nazionale delle Ricerche, Istituto di Fotonica e Nanotecnologie, piazza Leonardo da Vinci 32, 20133 Milano, Italy; lorenzo.spinelli@polimi.it; 4 Università degli Studi di Milano, Dipartimento di Medicina Veterinaria, via Celoria 10, 20133 Milano, Italy; michela.minero@unimi.it (M.M.); emanuela.dallacosta@unimi.it (E.D.C.); 5 Istituto Zooprofilattico Sperimentale dell’Abruzzo e del Molise G. Caporale, Campo Boario, 64100 Teramo, Italy; n.ferri@izs.it

**Keywords:** functional near-infrared spectroscopy (fNIRS), sheep, anticipatory behaviour, animal welfare, neuroimaging

## Abstract

**Simple Summary:**

Anticipatory behaviour to an oncoming food reward can be triggered via classical conditioning, implies the activation of neural networks, and may serve to study the emotional state of animals. This work aimed to investigate how the anticipatory response affects cerebral cortex activity in sheep. Eight ewes were conditioned to associate a neutral auditory stimulus (water bubble) to a food reward (maize grains). Then, sheep were trained to wait 15 s before accessing the food (anticipatory phase). Behavioural reaction and changes in cortical oxy-haemoglobin ([ΔO_2_Hb]) and deoxy-haemoglobin ([ΔHHb]) concentration were recorded by functional near infrared spectroscopy (fNIRS). During the anticipatory phase, sheep increased their active behaviour together with a cortical activation (increase of [ΔO_2_Hb] and a decrease of [ΔHHb]) compared to baseline. Sheep showed a greater response of the right hemisphere compared to the left hemisphere, possibly indicating frustration. Behavioural and cortical changes observed during anticipation of a food reward reflect a learnt association and an increased arousal, but no clear emotional valence of the sheep subjective experience.

**Abstract:**

Anticipatory behaviour to an oncoming food reward can be triggered via classical conditioning, implies the activation of neural networks, and may serve to study the emotional state of animals. The aim of this study was to investigate how the anticipatory response to a food reward affects the cerebral cortex activity in sheep. Eight ewes from the same flock were trained to associate a neutral auditory stimulus (water bubble) to the presence of a food reward (maize grains). Once conditioned, sheep were trained to wait 15 s behind a gate before accessing a bucket with food (anticipation phase). For 6 days, sheep were submitted to two sessions of six consecutive trials each. Behavioural reaction was filmed and changes in cortical oxy- and deoxy-hemoglobin concentration ([ΔO_2_Hb] and [ΔHHb] respectively) following neuronal activation were recorded by functional near infrared spectroscopy (fNIRS). Compared to baseline, during the anticipation phase sheep increased their active behaviour, kept the head oriented to the gate (Wilcoxon’s signed rank test; *p* ≤ 0.001), and showed more asymmetric ear posture (Wilcoxon’s signed rank test; *p* ≤ 0.01), most likely reflecting a learnt association and an increased arousal. Results of trial-averaged [ΔO_2_Hb] and [ΔHHb] within individual sheep showed in almost every sheep a cortical activation during the anticipation phase (Student *T*-test; *p* ≤ 0.05). The sheep showed a greater response of the right hemisphere compared to the left hemisphere, possibly indicating a negative affective state, such as frustration. Behavioural and cortical changes observed during anticipation of a food reward reflect a learnt association and an increased arousal, but no clear emotional valence of the sheep subjective experience. Future work should take into consideration possible factors affecting the accurateness of measures, such as probe’s location and scalp vascularization.

## 1. Introduction

Anticipatory behaviour is a response elicited by the association between two stimuli (e.g., neutral sound and food reward) and it can be triggered via classical conditioning [1,2]. It represents a specific class of emotional behaviours since the anticipation of emotional events guides, and thus affects, the behaviour of the subject experiencing it [3]. The act of anticipating implies the activation of neural networks representing the cue that is integrated with the most adequate response [1] and this can be reflected in the related behavioural expressions. In animal welfare science, anticipation in classical conditioning is an easy and useful tool to assess the balance between positive and negative experiences [1]. In this view, anticipatory behaviour to a food reward has been considered a manifestation of a positive affective state. This model has been applied, for instance, to the study of the emotional state of lambs anticipating positive events, like food or opportunities to play [4,5]. Understanding how affective states are represented in the brain is one of the major challenges that modern neuroscience faces today [6]. Brain indicators, in turn, offer important new perspectives to animal welfare science, namely by allowing a better understanding of the nature and magnitude of feelings as well as their effects on welfare [7]. Smulders [8] highlighted how, in animal welfare science, a neuro-scientific approach would help in improving the measurement of animal’s affective experiences that with behavioural observations alone would not be possible. Considering this aspect, different imaging techniques have been applied to study brain function to date. Functional magnetic resonance imaging (fMRI) has been the most useful in expanding the knowledge on the human brain [9]. However, this technique is challenging when applied to animals. In particular, the reduced size of the fMRI scanner, the demand of dedicated helmets, and the need to sedate or train the animals to reduce movement artefacts seriously limits its application to few species and experimental scenarios.

In recent years, functional near-infrared spectroscopy (fNIRS) has been proposed as a useful technique to assess brain cortical responses in animals [10] because it has a higher tolerance to movement artefacts and it can be applied on unrestrained animals in their familiar environment. The fNIRS technique employs near-infrared light to non-invasively measure the concentration of oxygenated haemoglobin [O_2_Hb] and deoxy-genated haemoglobin [HHb] in the brain cortex. Like fMRI, fNIRS exploits the neurovascular coupling mechanism that is the relationship between neural activity and the local change in cerebral blood flow (CBF). An increase in neural activity initially determines an increase in oxygen consumption and then it is accompanied by an increase in regional CBF. The overall effect is the so called haemodynamic response, that is, a net increase in the amount of [O_2_Hb] and a net decrease of [HHb] [11]. The neurovascular coupling is the driving mechanism that links behaviour (and more, in general, the response to sensorimotor and/or cognitive stimuli) to changes in the concentration of O_2_Hb and HHb in the cerebral cortex [11,12]. The fNIRS technique is non-invasive, safe, portable, and tolerant to motion artefacts, and it has been widely and successfully employed in several human studies (see, for example, [13]). Recently, fNIRS studies have also been carried out with wireless and miniaturized devices in free-moving domestic animals (see the recent review by [14]). fNIRS imaging was able to monitor changes in cerebral hemodynamic activity in animals, however, the effects of stimuli on the emotional responses of sheep were sometimes inconsistent among studies. Using an fNIRS device, Muehlemann and colleagues measured brain cortex activity in sheep exposed to a positive and a sham stimulus [15,16,17]. During grooming (positive stimulus), a decrease in cerebral O_2_Hb was found and the authors concluded that the brain response was unlikely to reflect pure somatosensory information. In 2014, brain cortex activation was measured in sheep exposed to stimuli of different perceived valence (negative, intermediate, and positive) [16]; when sheep were exposed to the negative stimulus, a stronger decrease in [HHb] was found. The results found in sheep partly differ from the classic evoked changes in cerebral oxygenation and haemodynamic response due to increased brain activity (increase in the amount of [O_2_Hb] and decrease of [HHb]). As the number of studies performed using fNIRS on animals is still low, further research is needed to improve measurement accuracy and to understand possible reasons for differences between human and animal results, and to clarify how fNIRS can be applied to the study of sheep emotion and welfare. The aim of the present study was to investigate, using a mobile fNIRS device for measurement, the cerebral cortex activity in free moving sheep during the anticipatory response to a food reward.

## 2. Materials and Methods 

### 2.1. Animals and Housing

**Figure 1:**
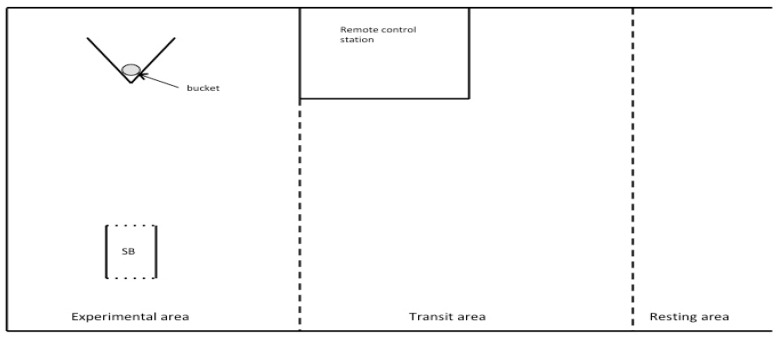
Schematic representation of the experimental pen (experimental area, transit area, and resting area). Dotted lines represent mobile fences; SB = Start box. Bucket with maize grains was placed behind a “V” shaped obstacle.

This study was conducted at the experimental farm of ‘Istituto Zooprofilattico Sperimentale dell’Abruzzo e del Molise’ (Italy). Fifteen no lactating nor gestating ewes (Sarda breed, 6 ± 1 year old) were randomly selected from the same flock. They had never been involved in any study before. Sheep were fed with hay twice a day (8 a.m. and 6 p.m.), and their diet was supplemented with a commercial concentrate (Mangimi Ariston Srl, Teramo, Italy; 250–300 g/ewe). All animals had free access to water and straw was provided for bedding. One month before the starting of the experiment, sheep were moved to the experimental pen. The pen was constituted by different areas: Experimental (16 m^2^), transit (20 m^2^), and resting (45 m^2^) (Figure 1). A ‘remote control station’ area was included. There, researchers controlled the start box (SB) gate (Figure 1) and the fNIRS probe without being visible to the animals. The access to the different areas was controlled by mobile fences.

### 2.2. Training Phase

**Figure 2:**
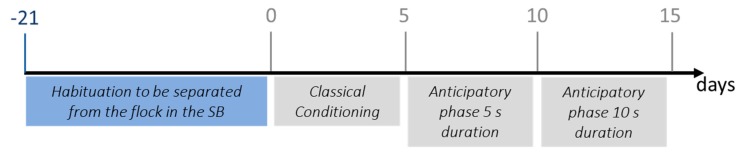
Schematic representation of the training phase: Days −21 to −1 (in blue) correspond to the habituation to be separated from the flock in starting box (SB). Days 0 to 15 (in grey) correspond to classical conditioning between the auditory stimulus and food reward (day 0–5) and increasing latency time from the end of the auditory stimulus to the opening of the gate of 5 s (day 6–10) and 10 s (day 11–15).

A schematic representation of the timeline of the training phase is reported in Figure 2. As a first step, sheep were trained to be separated from the conspecifics in the experimental area (isolation training). Over a three-week period, we gradually habituated the sheep to remain alone in the start box (SB). Initially, sheep entered the experimental area daily in groups of five animals and remained there for 30 min receiving a standard food reward (i.e., 100 g of maize grains) in a trough. After two days, the group size was reduced to three sheep and during the second week, sheep entered the area individually; a bucket with the food reward was positioned in place of the trough. During the third week, sheep were habituated to stay alone for 1 min in the SB, while being socially and visually isolated from the rest of the flock. The remaining sheep were kept in the resting area. Seven sheep that showed any high-pitched bleats or jumping attempts (stress signals) while in the SB after the third week of training were excluded from the sample; the remaining eight sheep underwent the second step of the training phase. 

As a second step, sheep were submitted to a classical conditioning paradigm to create an association between a neutral auditory stimulus (i.e., water bubble) and a food reward (maize grains). To do so, a neutral auditory stimulus of the duration of 5 s was presented when the sheep was in the SB. As soon as the stimulus ended, the gate was opened (with a remote control) and the sheep was allowed to reach the bucket with the food reward. In order to reach the bucket, the sheep had to detour a “V” obstacle (Figure 1) on its left or right side. Time to reach the bucket was recorded with a stopwatch. Within 5 days, all sheep reached the bucket in less than 10 s from the opening of the gate. In order to be able to evaluate the anticipatory response, we then gradually increased the latency time from the end of the auditory stimulus to the opening of the gate. Latency time increased from 5 s to 15 s (testing phase, see below). For 10 days, each sheep underwent two daily sessions composed of six consecutive trials each, according to the following schedule: 5 s from day 6 to 10 and 10 s from day 11 to 15. The sessions took place at least at two-hour intervals, after the sheep had been fasted for 2 h. 

### 2.3. Testing Phase 

The testing phase was carried out from day 16 to 21. fNIRS data were collected on day 21. Each sheep underwent two daily sessions, composed of six consecutive trials each. For each trial, data were recorded during:

- Baseline: The sheep was in the SB for 10 s;
- Auditory stimulus: Water bubble sound played for 5 s;
- Anticipation phase: 15 s duration after the auditory stimulus was played while the sheep was in the SB;
- Detouring: After opening the gate, the sheep was free to detour the obstacle and to reach the food reward.

#### 2.3.1. Behavioural Recordings

On days 16–21, video recordings of the sheep behaviour were collected with two cameras, one frontal and one rear of the focal subject. For each sheep, we recorded 72 trials (two sessions of six repetitions for six days). The ethogram used for behaviour analysis was adapted from previous studies [18,19], and is reported in Table 1.

Walking, scratching, and jumping were grouped as “active” behaviour. Videos were analysed using the software, Solomon Coder© (https://solomoncoder.com) [20], duration of behaviours was recorded, for each trial, during the baseline and anticipation phase. While in the detouring phase, the side used to detour the obstacle was recorded to then calculate the motor laterality index (MLI, in Statistical analysis). Finally, latency time to reach the bucket was directly recorded using a stopwatch. In order to take into account possible confounding effects of sudden head movement on fNIRS data collection, on day 21, we recorded also “head shaking” behaviour. 

#### 2.3.2. fNIRS Recordings and Analysis

**Figure 3:**
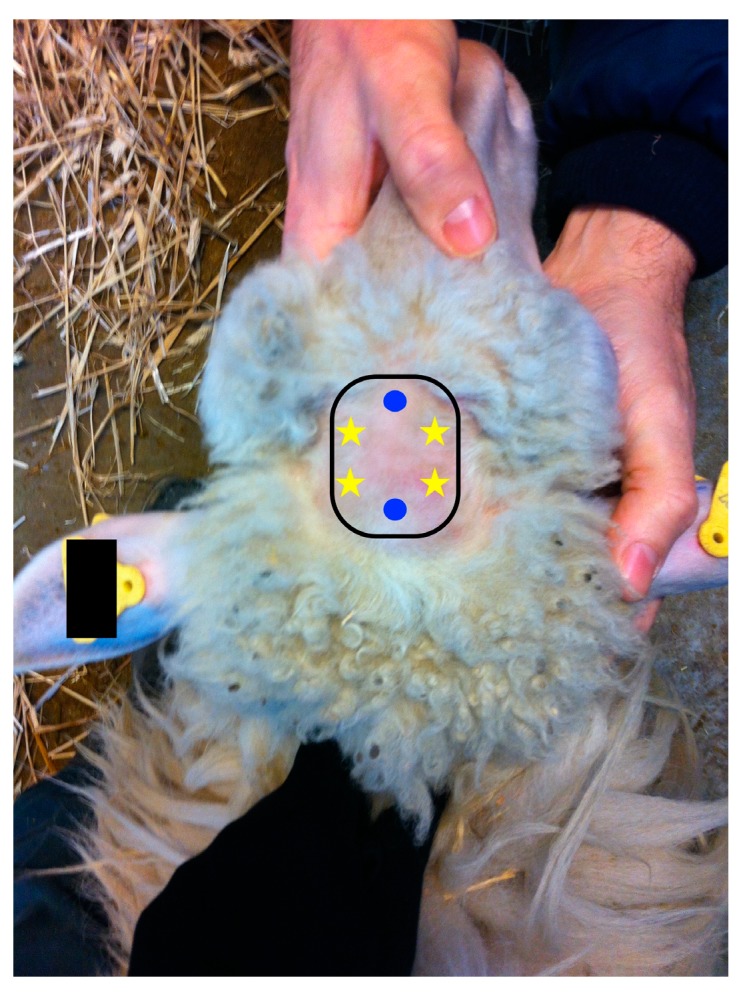
Schematic representation of the position of the fNIRS sensors (yellow stars represent the light sources and blue dots represents the detectors).

Two days before fNIRS recording, sheep were sheared on the head (an area of 2.5 cm × 3.5 cm) and habituated to wear a fake fNIRS sensor for 20 min (day 19 and 20) in the resting box, between the two daily testing sessions. After the habituation phase, no aversive behaviour to wearing the fNIRS probe was observed. On day 21, fNIRS data were collected by applying an fNIRS device (OxyPrem version 1.4, Division of Neonatology, Biomedical Optics Research Lab, University Hospital Zurich, Zurich, Switzerland) on the sheep head. The fNIRS sensor was attached to the forehead of the sheep’s depilated head, and fixed with a homemade cap. The sensor was composed of two detectors and four light sources at two different source-detector distances (1.5 cm and 2.5 cm), symmetrically arranged on the left and right hemisphere (Figure 3).

Light sources were sequentially activated, resulting in eight channels in total (i.e., all combinations of source and detector pair: Four channels at a 1.5 cm source detector distance and four channels at 2.5 cm). The device performed measurements at three wavelengths (760 nm, 805 nm, and 870 nm). Data were recorded using Tubis software (version 4.5, Division of Neonatology, Biomedical Optics Research Lab, University Hospital Zurich, Zurich, Switzerland). Signals were recorded with a sampling rate of 35 Hz. The fNIRS device measured raw light intensity values for the eight channels, including the signal and the backlight (i.e., the signal recorded when all light sources were switched off, therefore, depending on ambient light). In order to improve the signal-to-noise ratio (SNR), all data were first filtered (one dimensional median filter with a window of 5 s) and then the backlight was subtracted from the signal for each channel. Finally, we down sampled our data to 1 Hz according to Muehlemann et al. [15], which demonstrated that a 1 Hz sampling rate had sufficient bandwidth to detect the changes in [O_2_Hb] and [HHb] with reasonable computational demands and satisfactory SNR.

For each sheep, we recorded two sessions of six consecutive trials each. For each trial, we considered the baseline, auditory stimulus, and anticipation phases. We calculated [O_2_Hb] and [HHb] within the cortical tissue for the left and right hemisphere by applying the self-calibrating multi-distance approach [21], assuming values for the reduced scattering coefficient (0.52 mm^−1^ at 760 nm, 0.48 mm^−1^ at 805 nm, and 0.43 mm^−1^ at 870 nm) [22] and using the specific molar absorption coefficient for [O_2_Hb] (0.1518 mm^−1^ mM^−1^ at 760 nm, 0.2020 mm^−1^ mM^−1^ at 805 nm, 0.2800 mm^−1^ mM^−1^ at 870 nm) and for [HHb] (0.3153 mm^−1^ mM^−1^ at 760 nm, 0.2030 mm^−1^ mM^−1^ at 805 nm, 0.1881 mm^−1^ mM^−1^ at 870 nm) [23]. 

After calculating [O_2_Hb] and [HHb] for each hemisphere and for each sheep, the mean baseline concentrations were calculated in the last 5 s of the baseline to avoid possible effects of the previous trial and were subtracted from all recorded concentrations for each trial and each subject to obtain [ΔO_2_Hb] and [ΔHHb]), i.e., the changes in [O_2_Hb] and [HHb] with respect to the baseline. Furthermore, the trial-averaged [ΔO_2_Hb] and [ΔHHb] of each channel within individual subjects were calculated by averaging data across the trials. 

To quantify the hemodynamic response after the auditory stimulus, we then calculated the average of [ΔO_2_Hb] and [ΔHHb] over the last 15 s.

### 2.4. Statistical Analysis

Behavioural data were analysed with SPSS (version 20.1, IBM, Armonk, NY, USA). Descriptive statistics [mean, standard deviation (SD), and standard error of the mean (SEM)] were calculated. Based on the total length of the observation of the video recordings, durations of behaviours were calculated as the percentage of total observation time (proportional duration time). Data were tested for normality and homogeneity of variance using the Kolmogorov-Smirnov and Levene test, respectively. Behavioural data were not normally distributed; therefore, a non-parametric Wilcoxon’s signed rank test was used to identify differences in the proportional duration of different behaviour between the baseline and anticipation phases. Differences were considered to be statistically significant if *p* ≤ 0.05. Ear postures were excluded from analysis when sheep were wearing the fNIRS probe, as this could affect this specific behaviour.

In the detouring phase, to evaluate whether there was a side bias in detouring the obstacle, we calculated the individual lateralization with the binomial test. To assess the lateralization at the population level, we computed first a motor laterality index (MLI) for each subject with the following formula: MLI = (number of trials in which the sheep turned right/number of total trials)·100 [24]. Values of MLI lower than 50% indicated a preference for passing the obstacle on the left side, while values of MLI bigger than 50% indicated a preference for passing the obstacle on the right side. After that, we used MLI values and estimated departures from random choice (50%) by a median test. 

fNIRS data were assessed for a normal distribution and then analysed by the Student *T*-test for a paired sample. We compared mean values of [O_2_Hb] and [HHb] between the baseline and after the stimulus. We then analysed mean values of [O_2_Hb] and [HHb] after the stimulus between the two hemispheres.

To further analyse hemispheric asymmetry after the auditory stimulus, we used the mean values of [ΔO_2_Hb] and [ΔHHb] after the end the stimulus (15 s), to calculate the lateralized index response (LIR) for [ΔO_2_Hb] and [ΔHHb], respectively. For a given sheep, the LIR for [ΔO_2_Hb] and [ΔHHb] were evaluated according to the formula, LIR = (R − L)/(R + L), where R is either the mean [ΔO_2_Hb] or the mean [ΔHHb] over the last 15 s in the anticipatory phase for the right hemisphere, and L is either the mean [ΔO_2_Hb] or the mean [ΔHHb] over the last 15 s in the anticipatory phase for the left hemisphere. The LIR provides values in the range of [−1, +1]. A positive LIR indicated a greater response of the right hemisphere compared to left hemisphere, while a negative LIR indicated that the left hemisphere was more active than the right one. LIR = 0 represented no significant response after the stimulus (or equal response) in both hemispheres.

### 2.5. Ethical Note

This study was approved by the national ethical commission. Ministry of health authorization n° 457/2016-PR. 

## 3. Results

### 3.1. Behavioural Data

**Figure 4:**
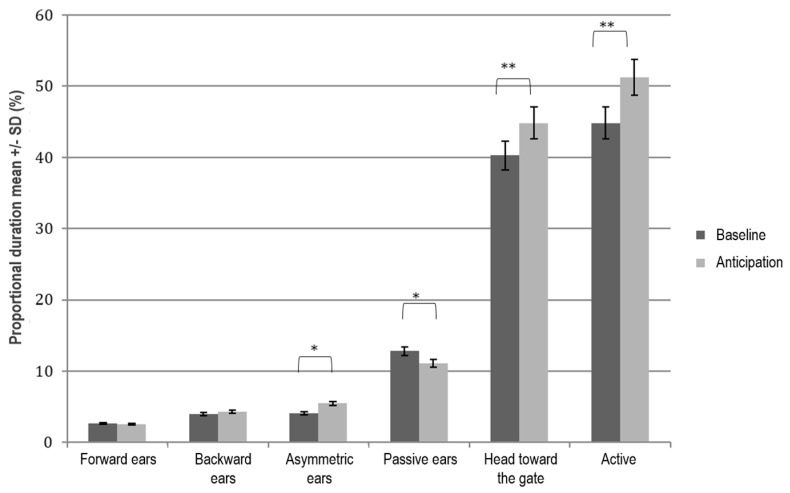
Proportion of time (%) sheep spent on behaviours (mean ± SD) during the baseline phase and anticipation phase while obtaining an anticipatory food reward (day 16–20). Wilcoxon’s signed rank test * *p* ≤ 0.01; ** *p* ≤ 0.001.

When the classical conditioning training was completed, the sheep learned the association between a neutral auditory stimulus (water bubble) and the presence of a food reward: The mean time (s) to detour the obstacle to reach the bucket decreased from 19 ± 11 s on the first day of training to 5 ± 2.6 s on the fifth day. Significant differences in behaviour were found between the baseline and anticipation phase (day 16–20), as reported in Figure 4. 

Sheep showed an increase of active behaviour and kept their head oriented to the gate more often in the anticipation phase compared to baseline (Wilcoxon’s signed rank test, *p* ≤ 0.001). Regarding ear posture, during the anticipation phase, sheep showed an increase of asymmetric ears and a decrease of passive ears compared to the baseline (Wilcoxon’s signed rank test, *p* ≤ 0.01).

During fNIRS recordings (day 21), we found similar behavioural results: In the anticipation phase, sheep were more active (51.2% ± 24.5) and kept their head oriented to the gate more often (44.8% ± 21.5) compared to the baseline, with 44.8% (±27.2) and 40.3% (±23.9), respectively (Wilcoxon’s signed rank test; *p* < 0.001). No head shaking was detected. We did not consider ear postures, as wearing the probe could affect them. 

At the population level, sheep did not show any difference in detouring the obstacle either on the left or right side. At the individual level, four sheep showed a preference to detour the obstacle on the right side (Binomial test, *p* < 0.01) and four sheep on the left side (Binomial test, *p* < 0.01).

### 3.2. Changes in Oxy- and Deoxy-Genated Haemoglobin in the Cerebral Cortex

**Figure 5:**
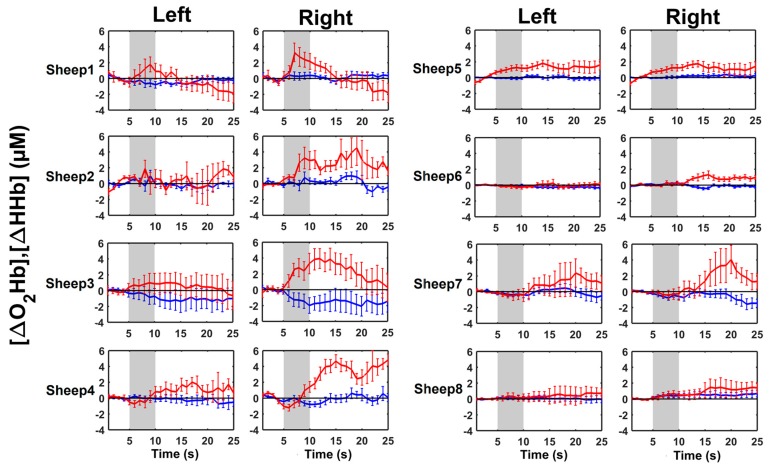
Trial-averaged oxygenated haemoglobin change [ΔO_2_Hb] (red lines) and deoxygenated haemoglobin change [ΔHHb] (blue lines), in µM, with respect to the baseline (0 s–5 s), as well as their standard deviations for each sheep in the left and right hemisphere. In every plot, the auditory stimulus (5 s–10 s) is marked as the grey area, followed by the anticipation phase (10 s–25 s). The horizontal black line in every sub-figure indicates the zero values.

The sheep were habituated to wear the fNIRS device, as we did not detect any head shaking during fNIRS recordings. From the results of the trial-averaged [ΔO_2_Hb] and [ΔHHb] within individual sheep, in almost every sheep, we observed a cortical activation in the anticipation phase. As it can be visually observed from Figure 5, four subjects (sheep 1, 3, 4, and 7) showed an increase of [ΔO_2_Hb] and a decrease of [ΔHHb] in both hemispheres. A bilateral increase in [ΔO_2_Hb] was observed also in two subjects (sheep 5 and 8), but without significant changes in [ΔHHb]. The remaining two subjects (sheep 2 and 6) showed an increase in [ΔO_2_Hb] in the right hemisphere only, without any changes for both [ΔO_2_Hb] and [ΔHHb] in the left hemisphere. 

Table 2 shows the individuals *p*-values for [ΔO_2_Hb] and [ΔHHb] in both hemispheres. In particular, we found 12 cases with *p* ≤ 0.05 for [ΔO_2_Hb] while only five cases for [ΔHHb]. The *p*-values confirmed the visual analysis of results reported in Figure 5, with the only exception of sheep 6, for which [ΔHHb] in the left hemisphere was characterized by a statistically significant (*p* < 0.05) change, but with a very small amplitude as compared to other sheep. Most of the LIR values for [ΔO_2_Hb] and [ΔHHb] were positive, indicating a greater response of the right hemisphere in the anticipation phase compared to the left one.

**Figure 6:**
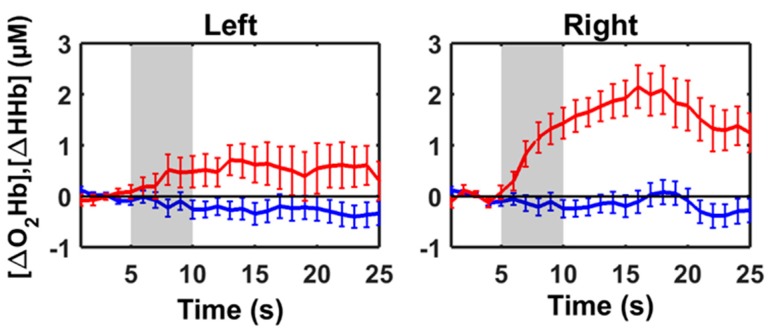
Group-averaged hemodynamic response and standard deviation of eight sheep for the left and right hemisphere. The plots (left and right) display the group-averaged oxygenated haemoglobin change [ΔO_2_Hb] (red lines) and deoxygenated haemoglobin change [ΔHHb] (blue lines), in μM, with respect to the baseline (0 s−5 s) of the left and right hemispheres. In every plot, the auditory stimulus (5 s−10 s) is marked as the grey area, followed by the anticipation phase (10 s−25 s). The horizontal black line in every sub-figure indicates the zero values.

The average result across all the sheep (group-averaged) showed an increasing of [ΔO_2_Hb] and a slight decrease of [ΔHHb] after the stimulus in both hemispheres (Figure 6). Student *T*-test for paired sample *p*-values of comparisons between the baseline and anticipatory phase were: [ΔO_2_Hb] 3.84 × 10^−9^ for the left hemisphere and 3.01 × 10^−10^ for the right hemisphere; [ΔHHb] 4.16 × 10^−7^ for the left hemisphere and 0.0316 for the right hemisphere. The LIR value for [ΔO_2_Hb] was + 0.50 and for [ΔHHb] was −0.28. 

## 4. Discussion

To better understand how the anticipatory response of free-moving sheep to a food reward affected the cortex activity, we integrated behavioural measurements (anticipatory response to a food reward, trained through classical conditioning) and measurement of cerebral cortex activity by means of the fNIRS technique. We report three major findings. Firstly, after the auditory stimulus, sheep showed more active behaviour and asymmetric ear posture. Secondly, we observed a cortical activation with a bilateral increase in [ΔO_2_Hb] in most sheep anticipating a food reward. Thirdly, we found a greater hemodynamic response of the right hemisphere compared to the left hemisphere.

The anticipation to an oncoming food reward is an appetitive classical conditioning response [25]. We found that sheep were more active when anticipating the upcoming event. The changes in behaviour most likely reflected a learnt association between the occurrence of a neutral sound and the oncoming food reward. It has been suggested that anticipation of a food reward is associated with a positive emotional state [2,26]. Anderson et al. [5] reported that lambs walked more when anticipating events considered positive and this was particularly evident when a food reward was used [5]. However, an intensification of active behaviour per se does not give clear information about the emotional valence (i.e., positive or negative) of an experience and it should be considered mainly as an expression of increased arousal. In fact, subjective experiences have not only an emotional valence, but they also vary in activation or arousal, and both positive and negative affective states can be characterized by high arousal [27]. In our study, when anticipating an oncoming food reward, sheep showed more asymmetric ear posture. Previous studies reported that in sheep, ear movements could be linked with the emotional state [18,28]; however, findings about the related emotional valence show some inconsistency. Reefmann and colleagues [18] found that “the proportion of asymmetric ear postures was influenced by the sequence of testing of the different feeding treatments, with a higher proportion of asymmetric ear postures during the first exposure to different feeding treatments”; therefore, they could not draw conclusions on the emotional valence during anticipatory behaviour. Boissy et al. [28] found that sheep presented asymmetric ear posture when exposed to very sudden situations, likely to elicit surprise. In our case, it was not possible to clearly understand the emotional valence of the subjective experience of sheep by only looking at their behaviour, as the observed behaviour was likely to be associated with an increased arousal that can be linked with both positive expectation and frustration. We did not find any lateral preference in detouring the obstacle at the population level, however, side preferences were found at the individual level, with four sheep preferring to detour on the right side and the remaining four on the left side. Several studies have used behavioural tasks to assess lateralization in sheep, but until now, a consistent lateral preference across breeds was not proven in sheep [29]. 

During the fNIRS recording, no head shaking was detected, thus we can assume that no movement artefacts have affected the results. At the cerebral cortical level, we found that neural activity occurred in both hemispheres during the anticipation phase. We also found a lateralized response, with a greater activation of the right cortex in sheep anticipating a food reward. In their extended review, Leliveld et al. (2013) [30] reported that—based on the ‘emotional-valence’ hypothesis—a right-hemisphere dominance would be more related to negative emotional states (e.g., fear and aggression). On the contrary, a left-hemisphere dominance would be more related to positive emotional states (e.g., response to receiving a food reward). Still, it should be noted that most of the considered studies were based on motor expression of lateralisation rather than on data recorded directly at the central level (i.e., from the brain). The observed greater activation of the right cortex could possibly be associated with a negative affective state, such as frustration. An alternative explanation could be that, during the anticipation phase, the cortex was involved mainly in attention processes. It has been reported that sheep, when undergoing an attention visual task, showed a faster response in the right hemisphere [31,32]. Recently, the fNIRS technique has been used in different animals: E.g., goats [10], dogs [33], and sheep [15,16,17], where the authors found some evidence that different positive and negative events had an effect on [O_2_Hb] and [HHb] in the brain cortex. In sheep, the decrease in [HHb] and an increase in the general activity and movement of the ears reflected a general activation in negative situations (i.e., intense localized pressure) [16]. In our study, we found a similar behavioural pattern (increased general activity and ear movement), but [HHb] only showed a minor decrease at the group level (and it was almost constant in four sheep). It is possible that sheep anticipating a food reward were in a differently valenced emotional state, but the discrepancy of results among different studies could also be related to different experimental settings. 

The small sample size was one of the major limitations of the current study. Future work should focus on a larger number of animals and include also a control group undergoing a neutral stimulus (a sound not associated with an outcome) and a negative control (a sound associated with a negative consequence). Nonetheless, it is important to emphasize that if the hemodynamic response was attributable only to a somatosensory reaction to the sound played, an equal response in both hemispheres would probably have been found. Another shortcoming of this study is that sheep could have learnt the sequence of the testing and started showing some anticipatory behaviour before the conditioned stimulus was played.

The fNIRS technique is still an innovative approach for studying cortex activity in farming animals. In the future, it will be necessary to improve the accurateness of the probe’s location, like in human studies, where more accessories (i.e., caps) and neuroanatomical information (e.g., anatomical and functional atlas) are available. Furthermore, to improve the signal quality, it will be useful to use shorter distances (e.g., 1 cm) to better assess the scalp vascularization, while assessing the cortex with longer distances (e.g., 3 cm). This will help to avoid confounding results as the more superficial hemodynamic events could interfere with the cortex recording. The potential and limits of this technique have been reported by [13] and [34]. It must also be noted that most of the cited studies in animals have assumed to record cortex activity in the frontal or prefrontal area, however, this needs to be established with other more in-depth measures.

Combining data from cortex activity and behavioural observations can provide a more in-depth evaluation of their expression and more specific conclusions to be made. However, it is currently still difficult to compare the brain function in different species. Animals might share brain areas, circuits, and pathways that mediate perceptual, visual, auditory, somatosensory, and other processes or, reversely, they could share homologous behaviours, possibly managed by different cytoarchitectonic areas [35]. Because subjective experiences are generated and processed by the brain, activity in the relevant cerebral areas might represent emotions more accurately than any other physiological and behavioural indicators [15].

## 5. Conclusions

Increased activity and changes in oxy- and deoxy-genated haemoglobin in the cerebral cortex were measured during anticipation of a food reward and reflected a learnt association between the occurrence of a neutral sound and the reward. The anticipatory behaviour shown might be related to increased arousal while the observed greater activation of the right cortex could possibly be associated with attention processes or frustration. Although this study was limited by the number of subjects involved, a neuroscientific approach is emerging in the animal welfare science, and fNIRS represents a non-invasive method that could contribute to obtaining meaningful information. As the studies employing fNIRS technology in animals have led to somehow inconsistent conclusions, it is important that future works take into consideration possible factors affecting the accurateness of measures, such as the probe’s location and scalp vascularization. 

## Figures and Tables

**Table 1:** Ethogram of recorded behaviours.

Behaviour	Description
Walking ^1^	At least two upper limbs and body moving in any direction
Jumping ^1^	The two anterior limbs up
Scratching ^1^	Rasp the floor or the wall with limb
Head toward the gate	Head pointed to the opening gate (direction of food reward)
Forward ears	Ears toward the front at an angle of more than 30° from the perpendicular
Backward ears	Ears toward the back at more than 30° from the perpendicular
Asymmetric ears	Ear tips are oriented in opposite directions
Passive ears	Ears hanging down loosely and dangling along with any head movement
Head shaking ^2^	Sudden movements of the head either up/down or left/right

^1^ Walking, scratching, and jumping were grouped as “active” behaviour. ^2^ Head shaking was collected only on day 21.

**Table 2:** *p*-values of paired sample Student *T*-test comparing the hemodynamic response (i.e., oxygenated haemoglobin change [ΔO_2_Hb] and deoxygenated haemoglobin change [ΔHHb]) in the baseline (0 s−5 s) and anticipatory phase (10 s−25 s) (both hemispheres of each sheep). Lateralized index response (LIR) values of [ΔO_2_Hb] and [ΔHHb] are reported for each sheep. LIR = (R − L)/(R + L), where R (L) is the mean [ΔO_2_Hb] or the mean [ΔHHb] over the anticipatory phase (10 s−25 s) for the right (left) hemisphere. The symbol ‘-’ indicates that the *p*-value is not statistically significant (*p* > 0.05).

	[ΔHHb]			[ΔO_2_Hb]		
	*p*-Value (L)	*p*-Value (R)	LIR	*p*-Value (L)	*p*-Value (R)	LIR
**Sheep 1**	2 × 10^−2^	-	−1	-	-	0
**Sheep 2**	-	-	0	-	3.43 × 10^−6^	+1
**Sheep 3**	8.44 × 10^−11^	8.19 × 10^−11^	0.16	5 × 10^−2^	7.01 × 10^−4^	0.67
**Sheep 4**	2 × 10^−2^	-	−1	5.07 × 10^−5^	8.23 × 10^−8^	0.52
**Sheep 5**	-	2.98 × 10^−6^	+1	7.73 × 10^−8^	1.47 × 10^−6^	0.20
**Sheep 6**	7.02 × 10^−8^	-	−1	-	7.26 × 10^−6^	+1
**Sheep 7**	-	2 × 10^−2^	+1	1.89 × 10^−4^	3.30 × 10^−3^	0.21
**Sheep 8**	-	7.72 × 10^−11^	+1	3.21 × 10^−5^	3.80 ×10^−6^	0.40
